# Effects of neoadjuvant trastuzumab, pertuzumab and palbociclib on Ki67 in HER2 and ER-positive breast cancer

**DOI:** 10.1038/s41523-021-00377-8

**Published:** 2022-01-10

**Authors:** Luca Gianni, Marco Colleoni, Giancarlo Bisagni, Mauro Mansutti, Claudio Zamagni, Lucia Del Mastro, Stefania Zambelli, Giampaolo Bianchini, Antonio Frassoldati, Ilaria Maffeis, Pinuccia Valagussa, Giuseppe Viale

**Affiliations:** 1 Fondazione Michelangelo, Milano, Italy; 2 IEO, European Institute of Oncology, IRCCS, Milano, Italy; 3 Azienda USL-IRCCS di Reggio Emilia, Reggio Emilia, Italy; 4 Department of Oncology, Azienda Sanitaria Universitaria Friuli Centrale, Udine, Italy; 5 Addarii Medical Oncology IRCCS Azienda Ospedaliero-universitaria di Bologna, Bologna, Italy; 6 IRCCS Ospedale Policlinico San Martino, UO Breast Unit, Genova, Italy; 7 Università di Genova, Dipartimento di Medicina Interna e Specialità Mediche (Di.M.I.), Genova, Italy; 8 Department of Medical Oncology, San Raffaele Scientific Institute, Milano, Italy; 9 Department of Oncology, Azienda Ospedaliero Universitaria di Ferrara - Arcispedale Sant’Anna, Ferrara, Italy; 10 IRCCS European Institute of Oncology, Milano, University of Milan, School of Medicine, Milano, Italy

**Keywords:** Targeted therapies, Breast cancer

## Abstract

The crosstalk between estrogen and HER2 receptors and cell-cycle regulation sustains resistance to endocrine therapy of HER2- and hormone receptor-positive breast cancer. We earlier reported that women with HER2 and ER-positive breast cancer receiving neoadjuvant dual HER2-block and palbociclib in the NA-PHER2 trial had Ki67 decrease and 27% pathological complete responses (pCR). We extended NA-PHER2 to Cohort B using dual HER2-block and palbociclib without fulvestrant and report here Ki67 drops at week-2 (mean change −25.7), at surgery (after 16 weeks, mean change −9.5), high objective response (88.5%) and pCR (19.2%). In Cohort C [Ki67 > 20% and HER2_low_ (IHC 1+/2+ without gene amplification)], women also received fulvestrant, had dramatic Ki67 drop at week 2 (−29.5) persisting at surgery (−19.3), and objective responses in 78.3%. In view of the favorable tolerability and of the efficacy-predictive value of Ki67 drop at week-2, the chemotherapy-free approach of NA-PHER2 deserves further investigation in HER2 and ER-positive breast cancer. The trial is registered with ClinicalTrials.gov, number NCT02530424.

## Introduction

It has been experimentally established that signaling pathways through the estrogen (ER) and the erbB2 (HER2) receptors converge on CDK1 and eventually to Rb checkpoint regulation^1–3^. We, therefore, postulated that concomitant triple block of ER, HER2, and Rb would result in enhanced antitumor activity that is especially attractive in ER+ and HER2+ breast carcinomas. In those now so called “triple-positive” tumors neoadjuvant trials consistently showed a lower rate of pathologic complete response (pCR) than in ER negative HER2+ tumors upon exposure to HER2-directed therapies in combination with chemotherapy^4–6^. In the NA-PHER2 trial we initially tested the effect of the concomitant triple block of the ER, HER2, and Rb on Ki67 labeling index and found a very rapid and significant decrease of proliferation at week 2, a clinical response rate in more than 95% of patients by week 16 of therapy, and a rate of pCR of 27% at surgery^7^.

In view of the good and almost preferential antitumor activity of palbociclib monotherapy in HER2+ cell lines^8^, a dual block of HER2 together with a block of RB1 may be adequate even without targeting the estrogen receptor. In addition, HER2 functional activation is a well-known mechanism of endocrine resistance in ER+/HER2 negative breast cancer^9^. We, therefore, expanded the NA-PHER2 study to two additional cohorts of patients. In cohort B we explored the effects of a concomitant block of HER2 and Rb in the absence of fulvestrant in patients with ER-positive and HER2-positive (IHC 3+ score or gene amplification by FISH) breast cancers. In cohort C the triple block of HER2, ER and Rb was studied in women with ER+ and HER2_low_ tumors (ICH 1+ or 2+ score and not amplified).

## Results

### Patients

**Fig. 1:**
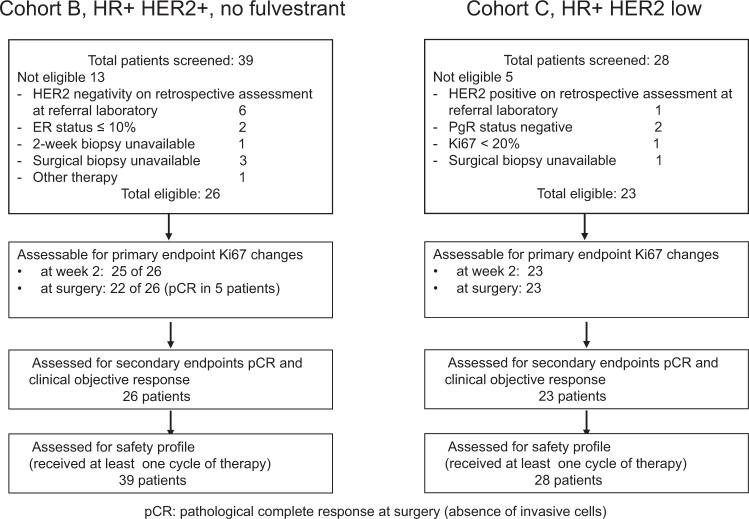
CONSORT diagram. Cohort B (left) and Cohort C (right) report the total number of screened patients, the number of eligible patients for the primary and secondary endpoints, and the number of patients assessed for safety.

Results for cohort A were already published^7^. A summary of patients’ disposition for cohorts B and C is outlined in Fig. 1.

**Table 1:** Main patient characteristics.

	Cohort B HR+ HER2+, no fulvestrant	Cohort C HR+ HER2 low
*Eligible patients*	26	23
Median age in year (range)	48.0 (29.0–86.0)	48.5 (35.0–79.0)
T stage		
cT1c	2 (7.7%)	2 (8.7%)
cT2	19 (73.1%)	18 (78.3%)
cT3	4 (15.4%)	3 (13.0%)
cT4b	1 (3.8%)	–
Nodal status		
cN0	15 (57.7%)	12 (52.2%)
cN1	11 (42.3%)	11 (47.8%)
Histology		
Ductal invasive	26 (100%)	22 (95.7%)
Lobular invasive	0	1 (4.3%)
PgR status		
Positive	17 (65.4%)	23 (100%)
Negative = 0	5 (19.2%)	0
HER2 +		
IHC 3+	15 (57.7%)	
Neu amplified	11 (42.3%)	

Overall, 39 patients were registered into cohort B between December 20, 2016, and October 27, 2017, and all received at least one cycle of planned therapy. The positive HER2 status was not confirmed in 6 cases, 2 additional cases had ER status ≤ 10%, mandatory biopsy was unavailable in 4, and one patient received another systemic therapy in the absence of disease progression. A total of 26 patients represent the eligible population of the study. Of the eligible patients, 25 had invasive cancer cells in tissue samples collected at week 2 after beginning study therapy, and 21 had invasive cancer cells in tissue samples collected at surgery. Table 1 describes the main patient characteristics of the eligible population. Of note, about 19% of the patients had clinical T3 or T4 and more than 50% had clinically palpable axillary lymph nodes.

A total of 28 patients were registered into cohort C between May 5, 2017, and February 16, 2018, and all received at least one cycle of planned therapy. HER2 status was not confirmed in 1 case, 2 additional cases had negative PgR status, in one case Ki67 value was <20%, and surgical biopsy was unavailable in one case. A total of 23 patients represent the eligible population of the study and all had invasive cancer cells in tissue samples collected at week 2 after beginning study therapy and at surgery. Table 1 describes the main patient characteristics of the eligible population. Of note, 13% of the patients had clinical T3 or T4 and more than 50% had no clinically palpable axillary lymph nodes.

### Effects on levels of KI67

**Fig. 2:**
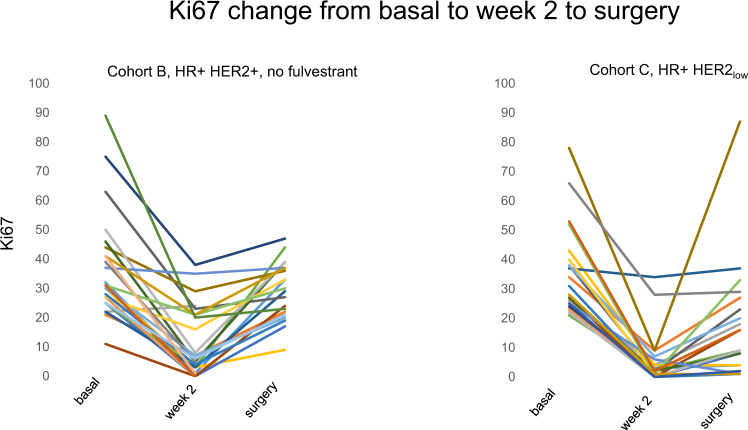
Individual levels of Ki67 at baseline, week 2 and surgery. In cohort B, in 1 of 26 cases at week 2 the tumor block failed to reveal the presence of invasive cells and a pCR was reached at surgery. At surgery, residual invasive disease was present in 22 patients while 5 patients achieved a pathological complete remission with no invasive cells in the breast and axilla. In cohort C, all the 23 cases presented invasive cells at week 2 and at surgery.

The primary goal of the trial was to study the effects of the drugs regimen on the levels of Ki67 at week 2 and/or at time of surgery. As illustrated in Fig. 2, the decrease of Ki67 was highly significant at both time intervals. In cohort B, at week 2 the geometric mean (±SD, *n* = 25) went from 33.4 ± 12.7 to 5.5 ± 12.4 (paired test *P* < 0·0001), and at time of surgery (*n* = 22) was 25.7 ± 9.7 (*P* = 0.033). In cohort C, in all 23 eligible patients, at week 2 the geometric mean went from 32.4 ± 19.9 to 2.6 ± 8.7 (*P* < 0.0001) and at time of surgery was 7.5 ± 20.4 (*P* < 0.001). Of interest, in 4 cases in the cohort B, Ki67 could not be measured due to lack of tumor cells at surgery. In Table 2, we report the decrease of Ki67 at week 2 and at surgery below 10% that was recently defined as threshold for sensitivity to endocrine therapy (ET) in tumors exposed to a brief course of ET^10^, and at or below 2.7%, the threshold of complete cell cycle arrest (CCCA)^11^. In cohort B 64% of tumors had a drop below 10% at week 2 and 9.1% at surgery, while CCCA was reached by 36% at week 2 and none at surgery. In cohort C more than 90% tumors showed a drop below 10% at week 2 and 56% at surgery, while CCCA was achieved in 65% at week 2 and 30% at surgery (Table 2). The geometric mean for apoptosis went from 1.0 ± 0.4 at baseline to 1.2 ± 0.4 at surgery in the 22 assessable cases in Cohort B (*P* = 0.94) and from 1.0 ± 0.4 to 0.4 ± 0.4 in the 23 cases of cohort C (*P* = 0.058).

**Table 2:** Decrease of Ki67 at week 2 and at surgery.

	Baseline (*N* = 26)	Week 2 (*N* = 25)	Surgery (*N* = 22)
*COHORT B*			
Mean (ranges)	37.1 (11–89)	11.2 (0–38)	27.8 (7–47)
Drop below 10%		64%	9%
Drop to less than 2.7%		36%	0
*HER2 IHC 3*+	(*N* = 15)	(*N* = 14)	(*N* = 13)
Mean (ranges)	43.3 (25–89)	12.7 (0–38)	30.1 (9–47)
Drop below 10%		57%	8%
Drop to less than 2.7%		35%	0
*HER2 neu amplified*	(*N* = 11)	(*N* = 11)	(*N* = 9)
Mean (ranges)	28.7 (11–44)	9.2 (0–29)	24.6 (7–36)
Drop below 10%		73%	11%
Drop to less than 2.7%		27%	0
	Baseline (*N* = 23)	Week 2 (*N* = 23)	Surgery (*N* = 23)
*COHORT C*			
Mean (ranges)	34.8 (21–78)	5.0 (0–34)	15.6 (1–87)
Drop below 10%		91%	56.5%
Drop to less than 2.7%		65%	30%

### Therapeutic activity

The therapeutic activity was analyzed during the entire duration of neoadjuvant therapy and at surgery. Clinical objective response immediately before surgery according to RECIST was reported in 23 of 26 patients or 88.5% (95% CI 69.8–97.6) in cohort B and in 18 of 23 patients or 78.3% (95% CI 56.3–92.5) in cohort C. (Tables 3 and 4). One patient in each cohort developed progressive disease while on treatment. At surgery, 5 of 26 patients (19.2%, 95% CI 6.6–39.4) had pCR in breast and nodes in cohort B, while no patients in cohort C achieved a pCR.

**Table 3:** Secondary endpoints: clinical response and pathological complete response rates in Cohort B (HER2+, ER+, no fulvestrant).

Eligible patients	26
Overall clinical response	23 (88.5%, 95% CI 69.8–97.6)
Complete response	9 (34.6%, 95% CI 17.2–55.7)
Partial response	14 (53.8%, 95% CI 33.4–73.4)
Stable disease	2 (7.7%, 95% CI 0.9–25.1)
Progressive disease	1 (3.8%, 95% CI 0.1–19.6)
Pathological complete response (absence of invasive cells in breast and axilla)	5 (19.2%, 95% CI 6.6–39.4)
*HER2 IHC 3+*	
Overall clinical response	13 (86.7%, 95% CI 59.6–98.3)
Complete response	6 (40.0%, 95% CI 16.3–67.7)
Partial response	7 (46.7%, 95% CI 21.3–73.4)
Stable disease	1 (6.7%, 95% CI 0.2–31.9)
Progressive disease	1 (6.7%, 95% CI 0.2–31.9)
Pathological complete response	3 (20.0%, 95% CI 4.3–48.1)
*HER2 neu amplified*	
Overall clinical response	10 (90.9%, 95% CI 58.7–99.8)
Complete response	3 (27.3%, 95% CI 8.0–61.0)
Partial response	7 (63.6%, 95% CI 30.8–89.1)
Stable disease	1 (9.1%, 95% CI 0.2–41.3)
Progressive disease	0
Pathological complete response	2 (18.2%, 95% CI 2.3–51.8)

*CI* confidence interval.

**Table 4:** Secondary endpoints: clinical response and pathological complete response rates in Cohort C (HR+ HER2_low_).

*Eligible patients*	23
Overall clinical response	18 (78.3%, 95% CI 56.3–92.5)
Complete response	3 (13.0%, 95% CI 2.8–33.6)
Partial response	15 (65.2%, 95% CI 42.7–83.6)
Stable disease	4 (17.4%, 95% CI 5.0–38.8)
Progressive disease	1 (4.3%, 95% CI 0.1–21.9)
Pathological complete response (absence of invasive cells in breast and axilla)	0

*CI* confidence interval.

### Safety

The profile of tolerability of the regimen was good. Overall, 2 treatment-related serious adverse events were recorded in the study, one in cohort B (1 of 39 or 2.6%, 95% CI 0.1–13.5) and 1 in cohort C (1 of 28 or 3.6%, 95% CI 0.1–18.3). As reported in Tables 5 and 6 for the two different cohorts, diarrhea was the most frequent adverse event. Grade 3 toxic effects were infrequent and reported for diarrhea, stomatitis, and hypersensitivity reactions. Neutropenia was the most frequent grade 3 event. Together with neutropenia, stomatitis and diarrhea were the most frequent reasons for delay or discontinuation of palbociclib. No deaths occurred during the study.

**Table 5:** Selected treatment emergent adverse events (TEAE, Safety population = 39 patients) in Cohort B (HR+ HER2+, no fulvestrant).

	% Any TEAE (95% CI)	% Grade 3 TEAE (95% CI)	
Neutropenia	64.1 (47.2−78.8)	30.8 (17.0−47.6)	
Diarrhea	59.0 (42.1−74.4)	7.7 (1.6−20.9)	
Mucosal inflammation	33.3 (19.1−50.2)	−	
Pyrexia	28.2 (15.0−44.9)	−	
Stomatitis	28.2 (15.0−44.9)	5.1 (0.6−17.3)	
Asthenia	20.5 (9.3−36.5)	−	
Fatigue	20.5 (9.3−36.5)	−	
Nausea	17.9 (7.5−33.5)	−	
Epistaxis	17.9 (7.5−33.5)	−	
Rash	17.9 (7.5−33.5)	−	
Vomiting	15.4 (5.9−30.5)	−	
Anemia	12.8 (4.3−27.4)	−	
Leukopenia	12.8 (4.3−27.4)	5.1 (0.6−17.3)	
Folliculitis	10.3 (2.9−24.2)	−	
Hemorrhoids	10.3 (2.9−24.2)	−	
ALT increased	7.7 (1.6−20.9)	2.6 (0.1−13.5)	
AST	7.7 (1.6−20.9)		−

*CI* confidence interval.

**Table 6:** Selected treatment emergent adverse events (TEAE, Safety population = 28 patients) in Cohort C (HR+ HER2_low_).

	% Any TEAE (95% CI)	% Grade 3 TEAE (95% CI)
Diarrhea	53.6 (33.9−72.5)	7.1 (0.9−23.5)
Neutropenia	50.0 (30.6−69.4)	42.9 (24.5−62.8)
Stomatitis	28.6 (13.2−48.7)	3.6 (0.1−18.3)
Mucosal inflammation	25.0 (10.7−44.9)	7.1 (0.9−23.5)
Pyrexia	25.0 (10.7−44.9)	−
Aphthous ulcer	14.3 (4.0−32.7)	−
Headache	14.3 (4.0−32.7)	−
Asthenia	10.7 (2.3−28.2)	−
Fatigue	10.7 (2.3−28.2)	−
Nausea	10.7 (2.3−28.2)	−
Leukopenia	10.7 (2.3−28.2)	3.6 (0.1−18.3)
Arthralgia	10.7 (2.3−28.2)	−
Dyspepsia	10.7 (2.3−28.2)	−
Hot flush	10.7 (2.3−28.2)	−
Hypertension	10.7 (2.3−28.2)	−
Anemia	7.1 (0.9−23.5)	−
ALT increased	7.1 (0.9−23.5)	−
AST increased	7.1 (0.9−23.5)	3.6 (0.1−18.3)
LVEF decrease	3.6 (0.1−18.3)	−

*CI* confidence interval.

## Discussion

The extended study of two additional cohorts of the NA-PHER2 trial showed in cohort B of HER2-amplified breast carcinomas that block of HER2 and cdk4/6 led to a rapid and major drop of Ki67 even without the use of estrogen receptor targeting in spite of estrogen receptor expression. We also showed that Ki67 rapidly and persistently dropped in cohort C of the NA-PHER2 trial, in which we applied the block of HER2, cdk4/6, and ER in women with HER2_low_ breast cancer characterized by centrally confirmed ER and PgR positive immunohistochemistry, and HER2 expression at 1+/2+ without gene amplification. In cohort B the block of HER2 and cdk4/6 without fulvestrant was associated with an 88.5% rate of objective response, 34.6% of complete clinical response and 19.2% pCR. In cohort C the clinical response was 78.3%, with 13% complete CR and no pCR. Tolerability and feasibility of the multidrug regimens without and with fulvestrant were good without observation of any major or limiting toxicity, as already described for cohort A of the study^7^.

A vast literature supports the value of Ki67 as a marker of potential sensitivity to endocrine therapy of HR+ breast carcinoma. Recently, the decrease of Ki67 at week 2 after a brief course of neoadjuvant perioperative endocrine therapy was validated in the large POETIC trial as predictor of long-term benefit of endocrine therapy in women with ER+ operable breast cancer^10^. A drop of Ki67 > 20% at day 15 of therapy with anastrozole was used in HER2+ tumors as reference to continue therapy with endocrine treatment and HER2 block in the PerElisa trial^12^. In the first cohort of NA-PHER2 we had shown a rapid, significant and very large drop of Ki67 at week 2 during neoadjuvant therapy with ET and block of HER2 and of cdk4/6^7^. A similar large effect on Ki67 was confirmed in cohort B of the study in ER+ and HER2+ tumors even without direct targeting of the estrogen receptor. The decision of testing the effects of dual block of HER2 and inhibition of cdk4/6 without ET was based on the well-known relative resistance of HER2+ tumors to ET^13–16^, and the demonstration that there exists a preferential sensitivity to palbociclib monotherapy in ER+ and HER2-amplified cell lines in the absence of ER targeting^17^. At week 2 all patients enrolled into cohort B had a decrease of Ki67, and 64% of them had a drop below 10%, the value that was recently validated as the threshold for sensitivity to ET at day 15 set in the POETIC trial^10^. Of note, at week 2 nine of the patients in cohort B (36%) had a drop of Ki67 to less than 2.7%, the threshold for Complete Cell Cycle Arrest (CCCA) that is a more stringent threshold indicating sensitivity to treatment in ER+ tumors^11^. The lack of endocrine therapy in the therapeutic regimen in cohort B of the NA-PHER2 may question the predictive relevance and the actual meaning of the Ki67 drop at week 2. However, the rate of CCCA is of the same order of magnitude of that recently reported for the cell cycle inhibitor abemaciclib without ET in women with ER+ breast cancer enrolled in the neo-MONARCH study^18^. Importantly, very high values of Ki67 were present at the baseline core biopsy in the cases reported here, with a mean value of 37%. This is in line with the findings in the POETIC trial of consistently higher values of Ki67 in HER2+ than HER2− breast cancer cases^10^. Overall, the relevant rate of objective clinical response and the observation of pathologic complete responses is suggestive of a possible association between observed therapeutic efficacy and extent of Ki67 modulation in cohort B. However, the lack of endocrine therapy in the regimen and the use of palbociclib that blocks cell cycle progression defines the link between Ki67 changes and prediction of treatment sensitivity only as a reasonable hypothesis that requires appropriate test in a prospective validation trial with event free survival as endpoint.

As already observed in cohort A of the study^7^, values of Ki67 in cohort B tended to rebound at time of surgery even though remaining lower than at baseline. A similar trend was already reported in other neoadjuvant trials of cdk 4/6 inhibitors (neoPalAna e neoMONARCH)^18,19^. In neoMONARCH the rebound was apparently linked to discontinuation of treatment with abemaciclib and anastrozole for more than 4 days^18^. In our study treatment was discontinued for a variable interval from end of planned therapy and time of surgery. In cohort B the rebound effect was more frequent and more pronounced than that reported in cohort A^7^ but also in cohort B we were unable to measure an effect of duration of drug-free interval on the rebound.

The dramatic drop of Ki67 at week 2 and the persisting drop at time of surgery were not associated with increased apoptosis, which was assessed at the same time intervals as Ki67. The data on apoptosis suggests that also in those cases in whom excellent clinical response was observed the efficacy was due to alternative mechanism of tumor shrinkage, including senescence which is a well-known mechanism of biologic effects of cdk4/6 inhibitors^20^.

In the present study we also showed that Ki67 rapidly and persistently dropped in cohort C of the NA-PHER2 trial, in which we applied the block of HER2, cdk4/6, and ER in women with HER2_low_ breast cancer that was characterized by ER and PR positive immunohistochemistry, and HER2 expression at 1+/2+ without gene amplification, as assessed at a central laboratory. Per protocol entry criteria, none of the patients in cohort C had a tumor with starting Ki67 below 20% at baseline. Interestingly, the drop of Ki67 was more persistent and profound than in cohort B and rebound was less frequent. Ki67 had values lower than 10% in 91.3% and 56.5.1% at week 2 and at surgery, respectively, and a Ki67 in the range of CCCA in 65.2% of cases at week 2 and 30.4% at surgery. The data in Ki67 changes are similar to those reported for abemaciclib and anastrozole in the neoMONARCH study^18^ although the rebound effect is again less pronounced and not linked to the duration of discontinuation of therapy until surgery. Also in cases with HER2_low_ tumors apoptosis was not apparently involved in the mechanism of observed therapeutic activity.

Overall, the reported additional cohorts of the NA-PHER2 study illustrate the course of Ki67 during a 16-weeks long therapy targeting cdk4/6 and HER2, and provide potentially useful information for the ongoing effort of defining novel and chemo-free treatments for women with HER2 positive breast cancer and hormone receptor positive breast cancer. The standard approach to date is based on targeting the HER2 receptor in combination with chemotherapy irrespective of the estrogen and progesterone receptor status of the tumor. In Cohort B of the study as in the previously reported Cohort A there is a relevant rate of Ki67 drop at week 2 that would qualify these cases as responsive if they were exposed to ET only as in the POETIC trial^10^. The course of Ki67 is accompanied by excellent clinical and even pathological response. Also the rate of progressive disease is in line with literature data focusing on neoadjuvant chemotherapy. The number of cases treated in the current study is small and exploratory, but the rate of objective response and of pCR is of the same order of magnitude than that reported for ER+ tumors exposed to dual block of HER2 and chemotherapy in the NeoSphere trial^4^. We think that the chemotherapy-free approach described in the NA-PHER2 deserves additional attention also in the light of the recent report of the KATHERINE trial that has documented the benefit of switching HER2-directed treatment from naked antibodies (mostly trastuzumab, but also trastuzumab and pertuzumab) to T-DM1 in cases of residual disease after neoadjuvant therapy^21^. Of note, the majority of patients with residual disease who entered the KATHERINE study had hormone receptor positive disease^21^. The observation is not only illustrating once again that HER2 and ER-positive breast cancers have a differential sensitivity to treatment with anti-HER2 therapy combined with chemotherapy, but also that such tumors deserve investigation of a more targeted approach that may avoid unnecessary toxicity.

The Cohort C in cases with HER2_low_ breast cancer deserves additional considerations. Patients were selected to have hormone receptors positive tumors including the expression of progesterone receptor. They also all had to have a Ki67 value of at least 20%. In brief, the patients were selected to have a luminal-B like type of breast cancer. It is of interest that a similarly selected group of patients with luminal B-like tumors enrolled in the ETNA trial and exposed to a 16 weeks-long chemotherapy with taxanes and anthracyclines had a rate of clinical response similar to that observed in the present cohort and a rate of pCR of only 10%^22^.

Our study has obvious limitations. The number of cases is small and limits the interpretation of the findings, and the therapeutic results were limited to clinical and pathological response without additional follow up for disease free survival after surgery. In addition, changes of Ki67 values during and after treatment as predictor of sensitivity were only validated for endocrine therapy and could have no such predictive value in the context of the regimens tested in the trial. However, the findings are consistent with the assumption that it may be worth testing the neoadjuvant application of a chemotherapy free approach to women with early breast cancer and HER2+ tumors while investigating in more detail the relative role and need for a pharmacologic block of the estrogen receptor, the cell cycle, the erbB-family of receptors and achieve an effective therapy tailored to the individual characteristics of the tumor. To move closer to such goal we are actively studying the molecular characteristics of the serial collection of tumors and blood of the NA-PHER2 trial. Such ongoing ancillary investigation may better inform the design of clinical trials to test the real value of the chemo-free regimens explored in the NA-PHER2 study.

## Methods

### Study design and patients

NA-PHER2 was an open-label, multicenter, exploratory phase II study performed in 7 Italian sites. The original protocol included only a cohort of patients (Cohort A) who received a 4-drug regimen named HPPF which included trastuzumab, pertuzumab, palbociclib, and fulvestrant. A protocol amendment included two additional cohorts of patients, named cohort B and cohort C. Most of the criteria and methods used in the study were already llustrated^7^.

In the section of the present report we will detail features that are characteristic of the two additional cohorts of the trial. The primary aim was to characterize, separately for each cohort, Ki67 changes from baseline before therapy at 2 weeks after starting therapy, and at surgery. Apoptosis changes were also assessed from baseline to surgery.

Secondary aims were: rate of pathological complete response (pCR) defined as absence of invasive cells in breast and axilla at surgery; clinical objective response rate at the end before surgery; and safety profile of the experimental therapy.

Patients eligibility required presence of previously untreated histologically confirmed unilateral invasive, HER2-positive (for cohort B) or HER2_low_ (for cohort C) and ER-positive (>10%) breast cancer. HER2 positive disease was always assessed according to ASCO/CAP guidelines in use at the time of the centralized review. Additional eligibility criteria for enrolment in cohort C were positive progesterone receptor (PgR) status and Ki67 > 20%. Patients had to consent to provide tumor tissues for centralized confirmation of HER2 and ER status and assessment of Ki67 values at required times. Other required characteristics were: age 18 years or older; ECOG performance status ≤ 1; tumor stage classified as cT1c to cT4a-d. Key exclusion criteria were metastatic disease, bilateral breast cancer, other malignancies, inadequate bone marrow or renal function, impaired liver function, impaired cardiac function, uncontrolled hypertension, pregnancy, and refusal to use contraception. HER2 status, ER, PgR and Ki67 had to be confirmed centrally.

The study was conducted according to Good Clinical Practice guidelines and the Declaration of Helsinki. All patients provided written informed consent. Approvals for the study protocol (and any modifications thereof) were obtained from independent ethics committees at each participating institution and relevant competent authority.

### Treatment and procedures

Within 5 days from registration, patients had to start protocol treatment, consisting of HPP for cohort B and HPPF for cohort C. Trastuzumab (H) was given at 8 mg/kg loading dose IV on cycle 1, and then at 6 mg/kg IV every 3 weeks. Pertuzumab (P) at 840 mg loading dose IV at cycle 1, and then at 420 mg IV every 3 weeks. H and P were given for total 6 administrations. Palbociclib (P) was given at 125 mg po q.d. for 21 days followed by one week rest (1 cycle to covering 4 weeks) and repeated for total 5 cycles. In cohort C fulvestrant (F) was given intra-muscle at 500 mg every 4 weeks and repeated for 5 times, with an additional 500 mg dose given 2 weeks after the first one. The total duration of neoadjuvant palbociclib (5 cycles every 4 weeks) and fulvestrant (5 administrations every 4 weeks plus the additional dose) was selected to match as closely as possible the total duration of the six 3-weekly planned administrations of H and P. Premenopausal women received an LHRH-agonist.

In presence of severe toxicity it was up to the investigators to decide for treatment discontinuation and immediate surgery. During neoadjuvant therapy physical, hematological and biochemical exams were performed before each treatment cycle and repeated before surgery. Clinical response in breast and nodes was assessed before surgery and was defined by modified RECIST criteria. Toxicity reported adverse events were categorized according to the NCI-CTCAE version 4.0. After neoadjuvant therapy patients underwent surgery and pCR was assessed by local pathologist according protocol guidelines.

Following surgery, additional adjuvant systemic therapy was given including chemotherapy (especially for patients with high tumor burden at baseline, positive axillary nodes at surgery, and at the Investigator’s discretion) plus standard HER2 treatment until completion of full 1 year if HER2 3+ or neu amplified and endocrine therapy according to local guidelines.

Post-surgery irradiation was recommended in all patients after breast-conserving surgery and in selected patients who underwent mastectomy according to international and local guidelines.

### Assessments

The primary endpoint was Ki67 expression level changes from baseline. Ki67 was scored in the central laboratory and reported in eCRF as the percentage of positively staining cells within the invasive margin in the examined area. Manual (visual) counting was performed, and percent of immunostained invasive tumor cells was assessed out of 500 cells or out of all the invasive tumor cells in the sections if less than 500 cells were present. A co-primary endpoint was to assess the changes in apoptosis from baseline to surgery. Changes in apoptosis were evaluated by counting apoptotic bodies at baseline and at surgery. Tumor samples were collected locally at all specified time points in all patients and stored at the central referral laboratory (European Institute of Oncology in Milano). All tumor blocks were labeled indicating the timing of the biopsy (baseline, week 2, surgery). The assessments of the primary endpoint was done centrally by one pathologist who was blinded for the outcome, and all the samples were assessed at the same time, including central assessment of HER2 and ER status on the baseline samples to confirm patient eligibility, with the exception of baseline assessment in Cohort C which was done at the time of registration. Kits used at the central laboratory for all the assessments were previously described^1^.

Other endpoints were the rate of pathological complete response at surgery, the rate of clinical objective response at the end of the neoadjuvant treatment and safety of the combination.

### Statistical analysis

There is no uniform consensus on the best cut-off values to discriminate between low and high scores of Ki67, and on the percentage changes that would qualify as clinically meaningful^23,24^, thus making difficult to derive a definite sample size. In light of this element, and also given the fully exploratory nature of the study, it was decided to determine the sample size upon feasibility and accrual rather than on formal statistical criteria.

To take into account the log-normal distribution usually followed by Ki67 measurements^7^ the geometric mean ± standard deviation were reported by time points. A *t*-test for paired samples on log-transformed data was used to detect statistically significant differences between scores at baseline and at 2 weeks, and between baseline and surgery. The *P* values reported are a probabilistic significance intended to measure the strength of the evidence that the reported results were not just a likely chance occurrence, not a biological one. Cases were assessable for Ki67 modifications only in the presence of positively staining cells within the invasive margin in the examined area. The same considerations and methods of analysis described for the Ki67 score apply to the changes in apoptosis score as well.

Therapeutic efficacy was assessed in all eligible patients. Clinical response in breast and nodes was assessed before surgery and was defined by modified RECIST criteria previously described^7^. The pathological complete response was defined as the absence of invasive disease in breast and axillary nodes at surgery. Exact 95% confidence intervals (Clopper–Pearson method) were calculated for clinical response and pathological complete response rates.

Toxicity reported adverse events were categorized according to the NCI-CTCAE version 4.0. Treatment-emergent adverse events (TEAE) were assessed by clinical examination, questioning for symptoms of toxicity, laboratory assessments (hematology and biochemistry), vital signs, at each physical examination during neoadjuvant therapy, before surgery and within 4–5 weeks after surgery. Cardiac examination with ECG and LVEF were to be repeated after the third cycle of neoadjuvant therapy and before surgery. Patients were analyzed according to the worst grade reported throughout the whole treatment period. TEAEs were assessed for all patients who received at least one treatment cycles, including patients who retrospectively failed to meet all eligibility criteria.

No sensitivity or interim analyses were planned. We analyzed data with SAS statistical software (version 9.4).

### Reporting summary

Further information on research design is available in the Nature Research Reporting Summary linked to this article.

## Supplementary information

Reporting summary

## Data Availability

The datasets generated during and/or analyzed during the current study are available from the corresponding author on reasonable request.
