# Supplementary material for: Effects of neoadjuvant trastuzumab, pertuzumab and palbociclib on Ki67 in HER2 and ER-positive breast cancer
Source: NPJ Breast Cancer. 2022 Jan 10;8:1. doi: 10.1038/s41523-021-00377-8 (PMC8748500; doi:10.1038/s41523-021-00377-8)
Supplement: Supplementary file 1 — Reporting summary [file 41523_2021_377_MOESM1_ESM.pdf]

nature portfolio

Corresponding author(s): Luca Gianni

Last updated by author(s): October 22, 2021

Reporting Summary

Nature Portfolio wishes to improve the reproducibility of the work that we publish. This form provides structure for consistency and transparency in reporting. For further information on Nature Portfolio policies, see our Editorial Policies and the Editorial Policy Checklist.

Statistics

For all statistical analyses, confirm that the following items are present in the figure legend, table legend, main text, or Methods section.

n/a	Confirmed	
☐	☒	The exact sample size (n) for each experimental group/condition, given as a discrete number and unit of measurement
☐	☒	A statement on whether measurements were taken from distinct samples or whether the same sample was measured repeatedly
☐	☒	The statistical test(s) used AND whether they are one- or two-sided<br>*Only common tests should be described solely by name; describe more complex techniques in the Methods section.*
☒	☐	A description of all covariates tested
☒	☐	A description of any assumptions or corrections, such as tests of normality and adjustment for multiple comparisons
☐	☒	A full description of the statistical parameters including central tendency (e.g. means) or other basic estimates (e.g. regression coefficient) AND variation (e.g. standard deviation) or associated estimates of uncertainty (e.g. confidence intervals)
☒	☐	For null hypothesis testing, the test statistic (e.g. F, t, r) with confidence intervals, effect sizes, degrees of freedom and P value noted<br>*Give P values as exact values whenever suitable.*
☒	☐	For Bayesian analysis, information on the choice of priors and Markov chain Monte Carlo settings
☒	☐	For hierarchical and complex designs, identification of the appropriate level for tests and full reporting of outcomes
☒	☐	Estimates of effect sizes (e.g. Cohen's d, Pearson's r), indicating how they were calculated

Our web collection on statistics for biologists contains articles on many of the points above.

Software and code

Policy information about availability of computer code

Data collection	The server and the OpenClinica application, which is the Clinical Data Management System (CDMS) envisaged for this study, will be installed in an instance for the creation and management of virtual servers, (name: AMB - NAP of Amsterdam - Michelangelo Tech - AMS1) supplied by Verizon Terremark.<br>Verizon Terremark was selected precisely for its characteristics of reliability and compliance with the standards and regulations relating to clinical trials (ICH-GCP and FDA CFR 21 part 11) and the supplier is able to produce - in relation to its own infrastructure - adequate documentation to demonstrate what is stated.
Data analysis	SAS (r) Proprietary Software 9.4 (TS1M6) Licensed to PROMEDITEC INNOVATION PER MICHELANGELO, Site 70137402.<br>Analytical products: SAS/STAT 15.1<br>NOTE: Additional host information: X64_10PRO WIN 10.0.18362 Workstation<br>Current version: 9.04.01M6P110718 Operating System: WX64_WKS

For manuscripts utilizing custom algorithms or software that are central to the research but not yet described in published literature, software must be made available to editors and reviewers. We strongly encourage code deposition in a community repository (e.g. GitHub). See the Nature Portfolio guidelines for submitting code & software for further information.

Data

Policy information about availability of data

All manuscripts must include a data availability statement. This statement should provide the following information, where applicable:

- Accession codes, unique identifiers, or web links for publicly available datasets
- A description of any restrictions on data availability
- For clinical datasets or third party data, please ensure that the statement adheres to our policy

The datasets generated during and/or analyzed during the current study are available from the corresponding author on reasonable request.

Field-specific reporting

Please select the one below that is the best fit for your research. If you are not sure, read the appropriate sections before making your selection.

☒ Life sciences ☐ Behavioural & social sciences ☐ Ecological, evolutionary & environmental sciences

For a reference copy of the document with all sections, see nature.com/documents/nr-reporting-summary-flat.pdf

Life sciences study design

All studies must disclose on these points even when the disclosure is negative.

Sample size	A minimum of 32 patients will be treated in each cohort of the current study in order to have at least 25 patients per cohort with fully adequate sampling for the assessment of the primary efficacy endpoints.<br><br>With regard to KI67, to date there is no uniform consensus on the best cut-off values to discriminate between low and high score and on the percentage change from baseline that would qualify for clinically meaningful result (Dowsett 2011, Pathmanathan 2013), thus making difficult to derive a definite sample size. In light of this consideration and also given the fully exploratory nature of the study, it was decided to determine the sample size upon feasibility, accrual and financial constraints rather than on formal statistical criteria.<br>A power analysis was performed on the change from baseline in Ki67 (it may apply as well to the change in apoptosis score), taking as fixed the size of 25 patients, with alpha=5% and with varying combinations of effect size, the mean of paired differences (either baseline vs. week 2 or baseline vs. measurement at surgery) and standard deviation (SD), both effect size and SD ranging from 1 to 10. Out of all possible combinations, the power turned out to be over the conventional value of 80% 73 times over 100.
Data exclusions	Registered patients who did not comply with the eligibility criteria were excluded from any efficacy analysis
Replication	No findings were replicated
Randomization	Registration will be done on a web-based e-CRF, accessible over a secured internet connection.<br>Patients who were candidates for enrollment into the study were evaluated for eligibility by the site investigator to ensure that the criteria detailed in the protocol were satisfied, and that the patient was eligible for participation in this clinical protocol. Once a patient fulfilled all the entry criteria, she can be registered into the protocol. The investigator or designee will then enter all patient eligibility data and information on e-CRF (Eligibility/Registration Form).<br>A unique patient number (UPN),without any reference to patient's initials and birth date, was automatically generated by EDC System for any patients registered on Eligibility/Registration Form.<br>Available paraffin-embedded tumor block taken at diagnostic biopsy were sent to the Central Laboratory for confirmation of HER2, ER and PgR eligibility and for assessment of Ki67 value and apoptosis
Blinding	Investigators were not blinded because the study was not a randomized one

Reporting for specific materials, systems and methods

We require information from authors about some types of materials, experimental systems and methods used in many studies. Here, indicate whether each material, system or method listed is relevant to your study. If you are not sure if a list item applies to your research, read the appropriate section before selecting a response.

Materials & experimental systems

n/a	Involved in the study
☐	☐ Antibodies
☐	☐ Eukaryotic cell lines
☐	☐ Palaeontology and archaeology
☐	☐ Animals and other organisms
☐	☐ Human research participants
☐	☐ Clinical data
☐	☐ Dual use research of concern

Methods

n/a	Involved in the study
☐	☐ ChIP-seq
☐	☐ Flow cytometry
☐	☐ MRI-based neuroimaging

Antibodies

Antibodies used — *Describe all antibodies used in the study; as applicable, provide supplier name, catalog number, clone name, and lot number.*

Validation — *Describe the validation of each primary antibody for the species and application, noting any validation statements on the manufacturer's website, relevant citations, antibody profiles in online databases, or data provided in the manuscript.*

Eukaryotic cell lines

Policy information about cell lines

Cell line source(s) — *State the source of each cell line used.*

Authentication — *Describe the authentication procedures for each cell line used OR declare that none of the cell lines used were authenticated.*

Mycoplasma contamination — *Confirm that all cell lines tested negative for mycoplasma contamination OR describe the results of the testing for mycoplasma contamination OR declare that the cell lines were not tested for mycoplasma contamination.*

Commonly misidentified lines (See ICLAC register) — *Name any commonly misidentified cell lines used in the study and provide a rationale for their use.*

Palaeontology and Archaeology

Specimen provenance — *Provide provenance information for specimens and describe permits that were obtained for the work (including the name of the issuing authority, the date of issue, and any identifying information). Permits should encompass collection and, where applicable, export.*

Specimen deposition — *Indicate where the specimens have been deposited to permit free access by other researchers.*

Dating methods — *If new dates are provided, describe how they were obtained (e.g. collection, storage, sample pretreatment and measurement), where they were obtained (i.e. lab name), the calibration program and the protocol for quality assurance OR state that no new dates are provided.*

☐ Tick this box to confirm that the raw and calibrated dates are available in the paper or in Supplementary Information.

Ethics oversight — *Identify the organization(s) that approved or provided guidance on the study protocol, OR state that no ethical approval or guidance was required and explain why not.*

Note that full information on the approval of the study protocol must also be provided in the manuscript.

Animals and other organisms

Policy information about studies involving animals; ARRIVE guidelines recommended for reporting animal research

Laboratory animals — *For laboratory animals, report species, strain, sex and age OR state that the study did not involve laboratory animals.*

Wild animals — *Provide details on animals observed in or captured in the field; report species, sex and age where possible. Describe how animals were caught and transported and what happened to captive animals after the study (if killed, explain why and describe method; if released, say where and when) OR state that the study did not involve wild animals.*

Field-collected samples — *For laboratory work with field-collected samples, describe all relevant parameters such as housing, maintenance, temperature, photoperiod and end-of-experiment protocol OR state that the study did not involve samples collected from the field.*

Ethics oversight — *Identify the organization(s) that approved or provided guidance on the study protocol, OR state that no ethical approval or guidance was required and explain why not.*

Note that full information on the approval of the study protocol must also be provided in the manuscript.

Human research participants

Policy information about studies involving human research participants

Population characteristics	Patients eligibility required presence of previously untreated histologically confirmed unilateral invasive, HER2-positive (for cohort B) or HER2low (for cohort C) and ER-positive (> 10%) breast cancer. HER2 positive disease was always assessed according to ASCO/CAP guidelines in use at the time of the centralized review. Additional eligibility criteria for enrolment in cohort C were positive progesterone receptor (PgR) status and Ki67 >20%.<br>Other required characteristics were: age 18 years or older; ECOG performance status ≤ 1; tumor stage classified as cT1c to cT4a-d. Key exclusion criteria were metastatic disease, bilateral breast cancer, other malignancies, inadequate bone marrow or renal function, impaired liver function, impaired cardiac function, uncontrolled hypertension, pregnancy, and refusal to use contraception. HER2 status, ER, PgR and Ki67 had to be confirmed centrally.
Recruitment	Patients who were candidates for enrollment into the study were evaluated for eligibility by the site investigator to ensure that the criteria detailed in the protocol were satisfied, and that the patient was eligible for participation in this clinical protocol. Once a patient fulfilled all the entry criteria, she can be registered into the protocol. The investigator or designee will then enter all patient eligibility data and information on e-CRF (Eligibility/Registration Form).<br>A unique patient number (UPN),without any reference to patient's initials and birth date, was automatically generated by EDC System for any patients registered on Eligibility/Registration Form.
Ethics oversight	First approval from the Independent Ethic Committee of the San Raffaele Scientific Institute in Milano, followed by approval of the Ethics Committes of al the participating sites

Note that full information on the approval of the study protocol must also be provided in the manuscript.

Clinical data

Policy information about clinical studies

All manuscripts should comply with the ICMJE guidelines for publication of clinical research and a completed CONSORT checklist must be included with all submissions.

Clinical trial registration	NCT02530424
Study protocol	The study protocol is available at www.fondazionemichelangelo.org
Data collection	Data were collected in an electronic CRF and for the present manuscript data were collected from December 27, 2016, to May 7, 2019.
Outcomes	The primary endpoint was Ki67 expression level changes from baseline. Ki67 was scored in the central laboratory and reported in eCRF as the percentage of positively staining cells within the invasive margin in the examined area. Manual (visual) counting was performed, and percent of immunostained invasive tumor cells was assessed out of 500 cells or out of all the invasive tumor cells in the sections if less than 500 cells were present. A co-primary endpoint was to assess the changes in apoptosis from baseline to surgery. Changes in apoptosis were evaluated by counting apoptotic bodies at baseline and at surgery. Tumor samples were collected locally at all specified time points in all patients and stored at the central referral laboratory (European Institute of Oncology in Milano). All tumor blocks were labeled indicating the timing of the biopsy (baseline, week 2, surgery). The assessments of the primary endpoint was done centrally by one pathologist who was blinded for the outcome, and all the samples were assessed at the same time. A secondary endpoint of this trial was the rate of pathologic Complete Response (pCR), determined as absence of invasive cells in breast and axilla (ypT0/ypTis, ypN0 according to AJCC classification). This secondary endpoint was done by the local pathologist who had to follow specific provided guidelines. For the purpose of this study, all clinical measurements were assessed by palpation of the breast and regional nodes. The objective response rate, defined as the proportion of patients who attained either a clinical complete response (cCR) or a partial response (cPR) during the study, was evaluated according to modified RECIST criteria (v 1.1), adapted to this protocol. Patients were assessed for adverse events by clinical examination, questioning for symptoms of toxicity, laboratory assessments, cardiac examination and vital signs.<br>Toxicities were assessed throughout the study according the National Cancer Institute (NCI) Common Terminology Criteria for Adverse Event (CTCAE) - version 4.0.<br>The analysis addressed the Treatment Emergent Adverse Events (TEAE) defined as all events with onset date posterior to the date of first treatment administration or started before the first study drug administration but worsening in severity during the treatment period. The incidence of TEAEs was grouped by System Organ Class (SOC) and by Preferred Term. Each patient was counted once for each SOC and/or for each preferred term, and in the analysis by CTCAE grade patients were analyzed according to the worst grade reported throughout the whole treatment period for that specific SOC/Preferred Term.<br>Adverse events (AEs) were coded by MedDRA dictionary, and the System Organ Class (SOC) and the Preferred Term (PT) are used for reporting purposes.

Dual use research of concern

Policy information about dual use research of concern

Hazards

Could the accidental, deliberate or reckless misuse of agents or technologies generated in the work, or the application of information presented in the manuscript, pose a threat to:

No	Yes	
☐	☐	Public health
☐	☐	National security
☐	☐	Crops and/or livestock
☐	☐	Ecosystems
☐	☐	Any other significant area

Experiments of concern

Does the work involve any of these experiments of concern:

No	Yes	
☐	☐	Demonstrate how to render a vaccine ineffective
☐	☐	Confer resistance to therapeutically useful antibiotics or antiviral agents
☐	☐	Enhance the virulence of a pathogen or render a nonpathogen virulent
☐	☐	Increase transmissibility of a pathogen
☐	☐	Alter the host range of a pathogen
☐	☐	Enable evasion of diagnostic/detection modalities
☐	☐	Enable the weaponization of a biological agent or toxin
☐	☐	Any other potentially harmful combination of experiments and agents

ChIP-seq

Data deposition

☐ Confirm that both raw and final processed data have been deposited in a public database such as GEO.

☐ Confirm that you have deposited or provided access to graph files (e.g. BED files) for the called peaks.

Data access links<br>*May remain private before publication.*	*For "Initial submission" or "Revised version" documents, provide reviewer access links. For your "Final submission" document, provide a link to the deposited data.*
Files in database submission	*Provide a list of all files available in the database submission.*
Genome browser session<br>(e.g. UCSC)	*Provide a link to an anonymized genome browser session for "Initial submission" and "Revised version" documents only, to enable peer review. Write "no longer applicable" for "Final submission" documents.*

Methodology

Replicates	*Describe the experimental replicates, specifying number, type and replicate agreement.*
Sequencing depth	*Describe the sequencing depth for each experiment, providing the total number of reads, uniquely mapped reads, length of reads and whether they were paired- or single-end.*
Antibodies	*Describe the antibodies used for the ChIP-seq experiments; as applicable, provide supplier name, catalog number, clone name, and lot number.*
Peak calling parameters	*Specify the command line program and parameters used for read mapping and peak calling, including the ChIP, control and index files used.*
Data quality	*Describe the methods used to ensure data quality in full detail, including how many peaks are at FDR 5% and above 5-fold enrichment.*
Software	*Describe the software used to collect and analyze the ChIP-seq data. For custom code that has been deposited into a community repository, provide accession details.*

Flow Cytometry

Plots

Confirm that:

- [ ] The axis labels state the marker and fluorochrome used (e.g. CD4-FITC).
- [ ] The axis scales are clearly visible. Include numbers along axes only for bottom left plot of group (a 'group' is an analysis of identical markers).
- [ ] All plots are contour plots with outliers or pseudocolor plots.
- [ ] A numerical value for number of cells or percentage (with statistics) is provided.

Methodology

Sample preparation	*Describe the sample preparation, detailing the biological source of the cells and any tissue processing steps used.*
Instrument	*Identify the instrument used for data collection, specifying make and model number.*
Software	*Describe the software used to collect and analyze the flow cytometry data. For custom code that has been deposited into a community repository, provide accession details.*
Cell population abundance	*Describe the abundance of the relevant cell populations within post-sort fractions, providing details on the purity of the samples and how it was determined.*
Gating strategy	*Describe the gating strategy used for all relevant experiments, specifying the preliminary FSC/SSC gates of the starting cell population, indicating where boundaries between "positive" and "negative" staining cell populations are defined.*

- [ ] Tick this box to confirm that a figure exemplifying the gating strategy is provided in the Supplementary Information.

Magnetic resonance imaging

Experimental design

Design type	*Indicate task or resting state; event-related or block design.*
Design specifications	*Specify the number of blocks, trials or experimental units per session and/or subject, and specify the length of each trial or block (if trials are blocked) and interval between trials.*
Behavioral performance measures	*State number and/or type of variables recorded (e.g. correct button press, response time) and what statistics were used to establish that the subjects were performing the task as expected (e.g. mean, range, and/or standard deviation across subjects).*

Acquisition

Imaging type(s)	*Specify: functional, structural, diffusion, perfusion.*
Field strength	*Specify in Tesla*
Sequence & imaging parameters	*Specify the pulse sequence type (gradient echo, spin echo, etc.), imaging type (EPI, spiral, etc.), field of view, matrix size, slice thickness, orientation and TE/TR/flip angle.*
Area of acquisition	*State whether a whole brain scan was used OR define the area of acquisition, describing how the region was determined.*

Diffusion MRI ☐ Used ☐ Not used

Preprocessing

Preprocessing software	*Provide detail on software version and revision number and on specific parameters (model/functions, brain extraction, segmentation, smoothing kernel size, etc.).*
Normalization	*If data were normalized/standardized, describe the approach(es): specify linear or non-linear and define image types used for transformation OR indicate that data were not normalized and explain rationale for lack of normalization.*
Normalization template	*Describe the template used for normalization/transformation, specifying subject space or group standardized space (e.g. original Talairach, MNI305, ICBM152) OR indicate that the data were not normalized.*
Noise and artifact removal	*Describe your procedure(s) for artifact and structured noise removal, specifying motion parameters, tissue signals and physiological signals (heart rate, respiration).*

Volume censoring | *Define your software and/or method and criteria for volume censoring, and state the extent of such censoring.*

Statistical modeling & inference

Model type and settings | *Specify type (mass univariate, multivariate, RSA, predictive, etc.) and describe essential details of the model at the first and second levels (e.g. fixed, random or mixed effects; drift or auto-correlation).*

Effect(s) tested | *Define precise effect in terms of the task or stimulus conditions instead of psychological concepts and indicate whether ANOVA or factorial designs were used.*

Specify type of analysis: ☐ Whole brain ☐ ROI-based ☐ Both

Statistic type for inference
(See Eklund et al. 2016) | *Specify voxel-wise or cluster-wise and report all relevant parameters for cluster-wise methods.*

Correction | *Describe the type of correction and how it is obtained for multiple comparisons (e.g. FWE, FDR, permutation or Monte Carlo).*

Models & analysis

n/a	Involved in the study
☐	☐ Functional and/or effective connectivity
☐	☐ Graph analysis
☐	☐ Multivariate modeling or predictive analysis

Functional and/or effective connectivity | *Report the measures of dependence used and the model details (e.g. Pearson correlation, partial correlation, mutual information).*

Graph analysis | *Report the dependent variable and connectivity measure, specifying weighted graph or binarized graph, subject- or group-level, and the global and/or node summaries used (e.g. clustering coefficient, efficiency, etc.).*

Multivariate modeling and predictive analysis | *Specify independent variables, features extraction and dimension reduction, model, training and evaluation metrics.*
